# Requirements for Proper Immunosuppressive Regimens to Limit Translational Failure of Cardiac Cell Therapy in Preclinical Large Animal Models

**DOI:** 10.1007/s12265-020-10035-2

**Published:** 2020-05-31

**Authors:** Evelyne J. Demkes, Simone Rijken, Mariusz K. Szymanski, Imo E. Hoefer, Joost P. G. Sluijter, Saskia C. A. de Jager

**Affiliations:** 1 Department of Cardiology, Laboratory of Experimental Cardiology, University Medical Center Utrecht, Heidelberglaan 100, 3508 GA Utrecht, The Netherlands; 2 UMC Utrecht Regenerative Medicine Center, Circulatory Health Laboratory, University Utrecht, University Medical Center Utrecht, Utrecht, The Netherlands; 3 Department of Cardiology, University Medical Center Utrecht, Utrecht, The Netherlands; 4 Central Diagnostic Laboratory, University Medical Center Utrecht, Utrecht, the Netherlands

**Keywords:** Immunosuppression, T-cells, Cell therapy, Xenogeneic, Heart failure, Preclinical, Animal models

## Abstract

Various cell-based therapies are currently investigated in an attempt to tackle the high morbidity and mortality associated with heart failure. The need for these therapies to move towards the clinic is pressing. Therefore, preclinical large animal studies that use non-autologous cells are needed to evaluate their potential. However, non-autologous cells are highly immunogenic and trigger immune rejection responses resulting in potential loss of efficacy. To overcome this issue, adequate immunosuppressive regimens are of imminent importance but clear guidelines are currently lacking. In this review, we assess the immunological barriers regarding non-autologous cell transplantation and immune modulation with immunosuppressive drugs. In addition, we provide recommendations with respect to immunosuppressive regimens in preclinical cardiac cell-replacement studies.

## Background

Ischemic heart disease, including myocardial infarction (MI), results in permanent and progressive loss of myocardial contractility. As a consequence of the reduced mortality after acute MI in the last decades [1], the prevalence of heart failure with reduced ejection fraction (HFrEF) is increasing. With more than 37.7 million patients, heart failure (HF), including HFrEF, is a substantial clinical problem and currently the fastest growing cardiovascular condition globally [2]. HF is characterized by the reduced ability of the heart to pump and/or fill with blood to support physiological circulation [3]. The only prevailing “curative” treatment for end-stage HF is heart transplantation. Unfortunately, there is a great discrepancy between supply and demand of donor hearts [4] resulting in many patients eligible for transplantation dying before receiving a matching donor organ [5]. Hence, current treatment options for HF are mostly aiming at reducing symptoms or delaying disease progression at best [4].

### Cell Sources for Cardiac Cell Transplantation

This lack of suitable treatment options emphasizes the need for new therapeutic strategies. Repair and regeneration of viable, functional myocardial tissue hold great promise. For this, several sources of cells have been explored, including “first-generation” cell types (e.g., bone marrow-derived mononuclear cells or mesenchymal stromal cells) of which currently phase 3 trials are ongoing [6]. However, “second-generation” cell types including cardiac-derived progenitor cells (e.g., cardiospheres, Sca-1+ cardiac progenitor cells) and pluripotent stem cells (e.g., ESCs and iPSCs)-cardiac derivatives exhibit higher reparative potential [7]. Currently, more advanced approaches with these cell sources are being developed, such as cell sheets [8–10], cardiac aggregates [11, 12], and engineered heart tissue [13, 14], to increase cell retention, survival, and boost therapeutic action.

### Immunogenicity of the Transplanted Cells in Preclinical Studies

Feasibility, safety, and efficacy of novel therapies have to be tested in relevant large animal models. Most often, xenogeneic cells are applied in such studies as human cell products are more interesting from a clinical translation perspective and autologous cell preparation is unrealistic regarding costs and labor intensity [15]. However, one of the biggest challenges to overcome is the host’s immunologic intolerance upon transplantation. Non-autologous cells are recognized by the host’s immune system as non-self, requiring immunosuppressive therapy to prevent rejection and thus loss of function. Many immunosuppression regimens have been reported with mixed effects on cell transplant survival and treatment efficacy. As most studies do not strictly monitor the level of immunosuppression and it remains unclear whether the used dose of immunosuppression is optimal and sufficient for transplant survival.

Therefore, the aim of this review is to evaluate immunosuppressive protocols for cardiac cell transplantation in preclinical large animal heart failure models to provide recommendations on the use of immunosuppression in preclinical studies.

## Cell Transplant Immunology

Immune cells continuously patrol the body to search for invading agents, differentiating between “self,” i.e., autologous and “non-self,” i.e., non-autologous, to protect integrity and health. Accordingly, transplantation of non-autologous cells can result in immune reactions with high probability of rejection. This clearance is primarily caused by acute cellular rejection driven by T cell alloantigen recognition.

**Fig. 1:**
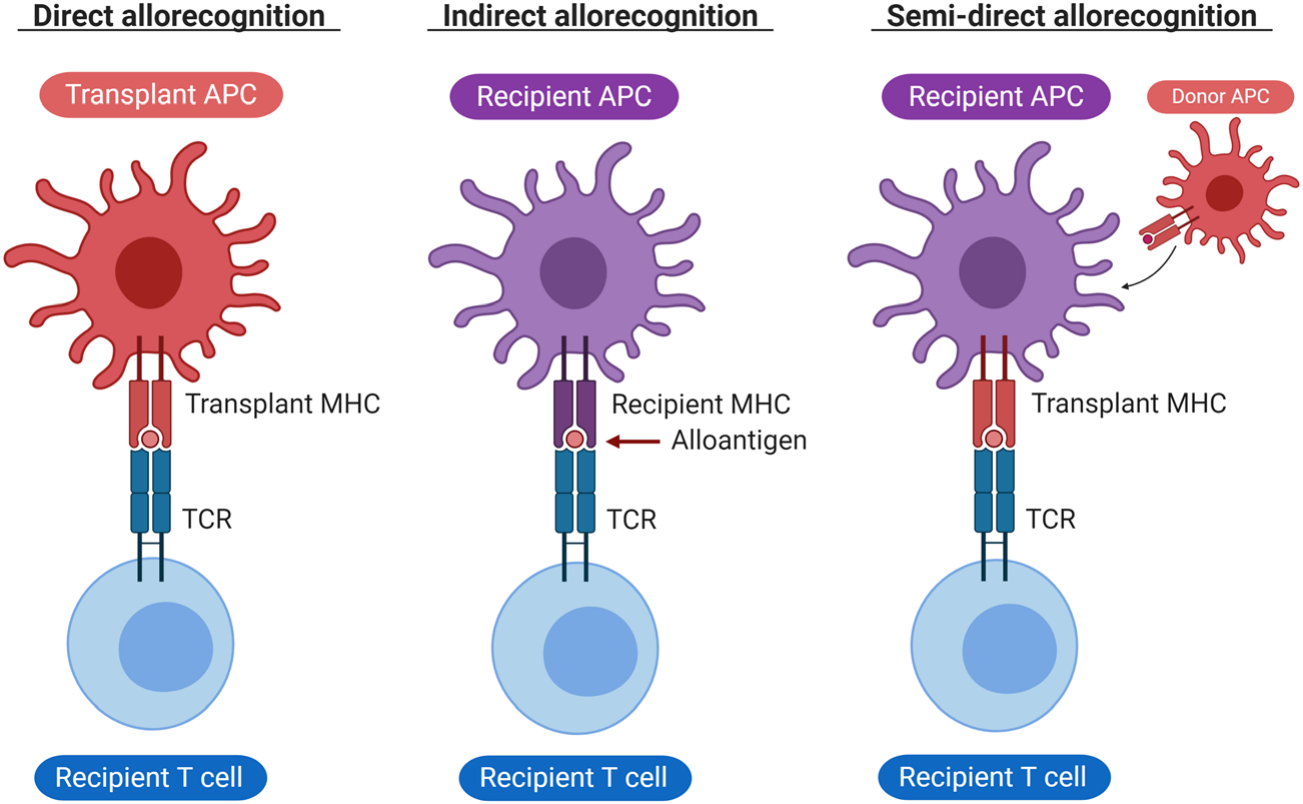
Three distinct ways for T cell allorecognition: direct, indirect, and semi-direct. In the direct pathway, transplant APC interacts directly with recipient T cells. In indirect recognition, recipient APCs present processed transplant peptides (alloantigen) to recipient T cells. In the semi-direct pathway, recipient APCs acquired transplant HLA that present peptides directly to recipient T cells. APCs, antigen-presenting cells; TCR, T cell receptor. Figure was created with Biorender.com

T cells recognize non-autologous cells by major histocompatibility complex (MHC) expression. MHCs are polymorphic cell surface glycoproteins that present peptide fragments, i.e., antigens, derived from self and foreign proteins. MHC class I molecules are expressed on almost all nucleated cells and present intracellular peptides to CD8+ cytotoxic T cells. MHC class II molecules are only expressed on antigen-presenting cells (APCs) and present extracellular proteins and pathogens to CD4+ T helper cells. A mismatch in MHC class or non-self-antigens presented by MHC molecules can be recognized via three distinct pathways: the indirect, direct, and semi-direct pathway (Fig. 1). In the indirect pathway, non-self-peptides are recognized by the T cell receptor (TCR) after they have been internalized and presented on the host APCs [16]. In the direct pathway, the TCR recognizes non-self MHC molecules with bound peptides on the surface of the transplanted cells or transplants APCs [16]. In semi-direct allorecognition, donor MHC-peptide complexes are captured by host APCs after MHC cross-presentation, which is the transfer of preformed functional peptide-MHC complexes from the surface of donor cells to recipient cells via cell-cell contact or through extracellular vesicles [17, 18]. After alloantigen recognition, T cell is activated by two signals: interaction between the TCR–CD3 complex with the MHC on the APC (signal 1) and interaction between co-stimulatory signals, such as CD28 on the T cell with CD80 and CD86 on the APC (signal 2) [19, 20]. Proliferation and polarization of the T cell require a third signal [20], which is established through downstream signaling pathways following T cell activation and secretion of cytokines by APCs.

Clinically, donor and recipient are matched as good as possible for blood group antigens and HLA to minimize the rejection risk [21]. Additionally, patients receive immunosuppressive drugs to further reduce immune responses. Our understanding of the complexity of inflammatory responses to xenografts is increasing progressively and a lot can be learned from the field of xenotransplantation [22]. With the development of humanized models by gene editing, human cells may be transplanted in animal models without the need for immunosuppression in the future. Although great progress is made in this area, the use of large transgenic animals is limited by the difficulties of genome-editing technologies, the complexity of generating healthy transgenic animals, costs, safety, and ethical issues [23]. Therefore, immunosuppressive therapies in preclinical animal studies yet remain of great importance.

## Immunosuppression for Prevention of Rejection

Preventing xeno-cell transplant rejection in large animal models as much as possible demands efficient immunosuppressive regimens. As T cell allorecognition is the main contributor to transplant rejection, clinical therapies targeting peripheral leukocytes are consequently effective in preventing acute rejection and improving long-term graft survival and patient outcomes [24].

**Fig. 2:**
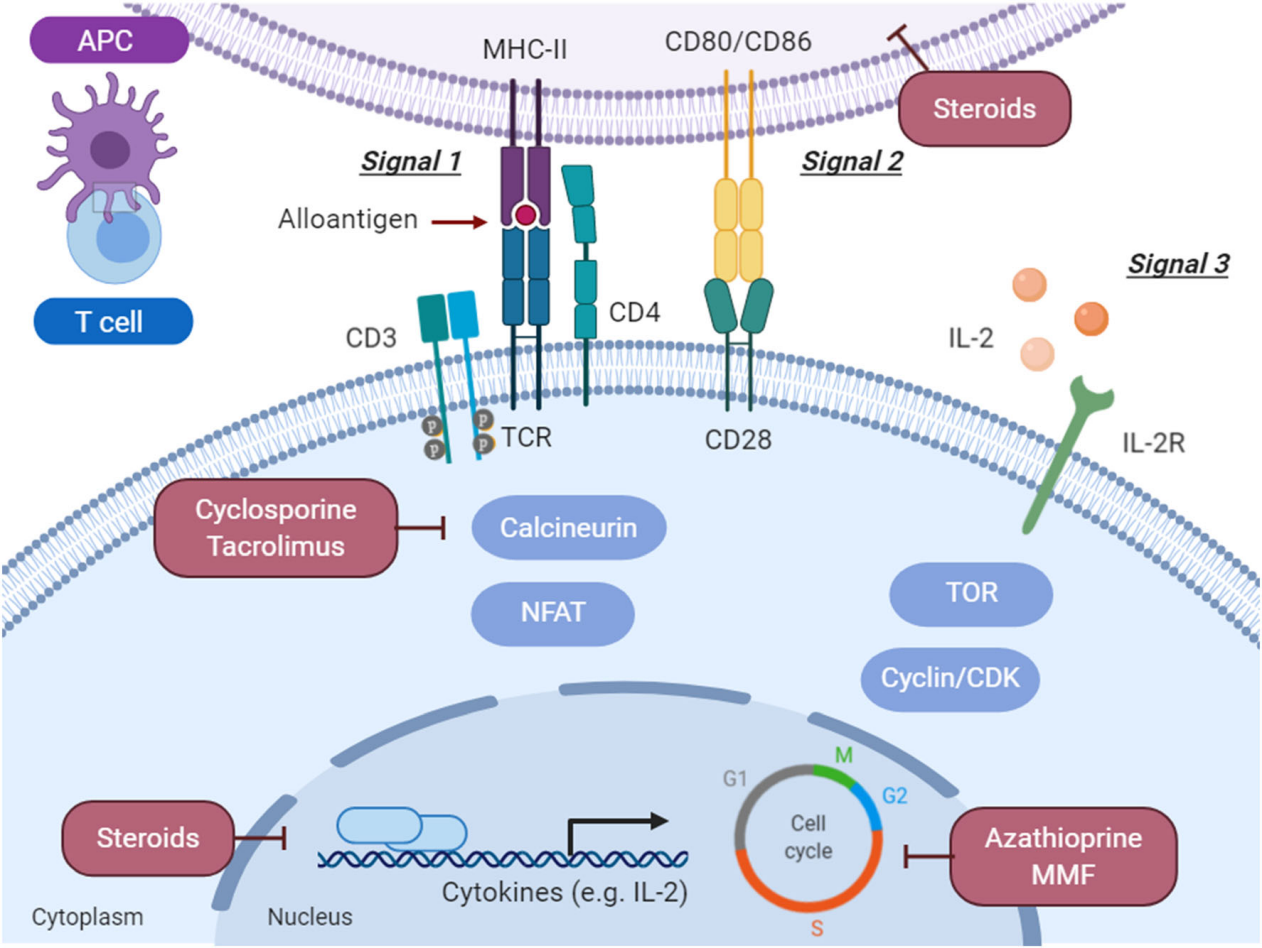
Immunosuppressive agents targeting signaling pathways for T cell activation, proliferation, and polarization. T cell activation results from alloantigen recognition via the T cell receptor (TCR)–CD3 complex with the MHC-II on the APC (signal 1) and a co-stimulatory signal from CD28-CD80/CD86 on the APC (signal 2). Stimulation by IL-2 (signal 3) results in cell proliferation and polarization. Immunosuppressive agents (shown in red boxes) exert their effects by inhibiting a number of different targets. G1 (first growth phase), S (synthesis of DNA), G2 (second growth phase), and M (cell division) represent the phases of the cell cycle. APC, antigen-presenting cell; CD, cluster of differentiation; CDK, cyclin-dependent kinase; IL-2, interleukin-2; IL-2R, interleukin-2 receptor; MHC, major histocompatibility complex; MMF, mycophenolate mofetil; NFAT, nuclear factor of activated T cells; TCR, T cell receptor; TOR, target of rapamycin protein. Figure was created with Biorender.com

Immunosuppressive treatment can be separated in induction (strong immunosuppression in early postoperative phase), maintenance (long-term prevention of acute and chronic rejection), and anti-rejection regimens (used to treat rejection). The added value of induction therapy is however being questioned, as no significant reduction of mortality, adverse events, infection, or cardiac allograft vasculopathy has been observed [25]. Clinical maintenance regimens apply high-intensity immunosuppression in the first weeks after surgery followed by decreasing doses. Such regimens are generally based on combinations of several drugs at lower doses to reduce the occurrence of unwanted side- and toxic effects. Most commonly, calcineurin inhibitors, anti-proliferative agents, and corticosteroids are combined for this purpose. For a detailed overview, we refer to Wiseman et al. [26]. Here, we provide a concise overview of common conventional immunosuppression agents and their mode of action as illustrated in Fig. 2.

### Calcineurin Inhibitors

Calcineurin inhibitors, such as cyclosporin A (CyA) and tacrolimus, form the cornerstone of immunosuppressive therapy in solid organ transplantation. Calcineurin plays a central role in signal transduction upon TCR-ligand binding (signal 1). Calcineurin dephosphorylates nuclear factor (NF) of activated T cells (NFAT), enabling NFAT translocation from the cytoplasm to the nucleus, where it leads to transcription of its target genes, including various pro-inflammatory cytokines required for T cell proliferation and polarization, and for providing B cell assistance [27]. CyA forms a complex with cyclophilin that subsequently binds to calcineurin thereby inhibiting its activation [28, 29]. Tacrolimus inhibits the calcineurin pathway one step upstream of cyclosporin, by binding to the immunophilin FK506 binding protein 12 (FKBP-12) and forming a complex of tacrolimus-FKBP-12, calmodulin, calcium, and calcineurin [28, 29].

Both inhibitors have similar effects on cytokine release and T cells but show different efficacy and side-effect profiles [28]. In vitro and in vivo, tacrolimus appeared more effective and associated with lower allograft rejection rate compared to CyA [30–33]. In addition, tacrolimus and CyA have different pharmacokinetic profiles with confounding factors like patient age, transplant type, and other medication influencing their plasma concentrations. Due to interpatient variability and narrow therapeutic index, determining optimal dosage to ensure sufficient immunosuppression and minimizing side effects is crucial. Therefore, monitoring of plasma levels has become a standard practice for patients receiving calcineurin inhibitors and more methods for routine monitoring are being developed [34].

A well-known side effect of both CyA and tacrolimus is nephrotoxicity [30]. Hypertension and hyperlipidemia have also been reported, but are more frequently observed for CyA. On the other hand, patients treated with tacrolimus are 2–3 times more likely to develop new-onset diabetes mellitus and this risk increases with higher dosages of tacrolimus [31]. Neurological side effects such as tremors are more common with tacrolimus while CyA causes cosmetic side effects, such as gum hyperplasia and abnormal hair growth [30, 31].

### Glucocorticoids

Glucocorticoids exert immunosuppressive effects by regulating gene expression that affects several players of the immune response, such as T and B cells, macrophages, eosinophils, and monocytes [26]. They are highly effective both in prevention and treatment of acute rejection and are therefore effective in post-transplantation management.

The most commonly used glucocorticoids for immunosuppression are prednisone and (methyl-) prednisolone. Prednisone is metabolized in the liver to its active form prednisolone, which binds with high affinity to glucocorticoid receptors in the cytoplasm of potential target cells [26]. Binding allows the glucocorticoid receptor complex to translocate to the cell nucleus where it inhibits transcription factors, such as NF-*κβ* and activator protein-1 [35]. This results in reduced production of a broad panel of pro-inflammatory cytokines, including IL-1, IL-2, IL-5, and TNF-α, adhesion molecules and chemotactic proteins.

Long-term glucocorticoid use can lead to serious side effects like osteoporosis [36], muscle atrophy [37], and multiple endocrine-, metabolic-, cardiovascular-, and dermatologic side effects (reviewed in [38]). Side effects may occur in over 90% of patients who take glucocorticoids for more than 60 days [39]. To reduce the probability of developing side effects upon long-term use, glucocorticoids are started in high dosage and are subsequently tapered to lower doses; often discontinued within 1-year after transplant.

### Anti-proliferative Immunosuppressive Drugs

Another way to achieve immunosuppression is by inhibiting proliferation and/or induces cell death. The most commonly used cytostatic and anti-proliferative compounds used to this end are azathioprine and mycophenolate mofetil (MMF).

Azathioprine belongs to the family of thiopurine compounds that structurally resemble endogenous purines. As a prodrug, it is rapidly converted by plasma esterases or non-enzymatically by glutathione to 6-mercaptopurine, and finally to several metabolites, including 6-thioguanine nucleotides (6-TGNs) [40]. TNGs are incorporated into DNA and RNA, thereby inhibiting cell cycle progression [41, 42]. Apart from that, azathioprine is also able to convert the co-stimulatory signal of CD28 into an apoptotic signal by modulating Rac1 activity and suppresses APC-T cell conjugation, thereby preventing induction of T cell activation necessary in effective immune response [43, 44]. Mycophenolate mofetil (MMF) inhibits a key enzyme in guanine nucleotide synthesis: inosine monophosphate dehydrogenase. While other cell types can synthesize purines via alternative pathways, proliferating lymphocytes are depending on this enzyme for DNA replication [45], which makes MMF selective for lymphocytes.

Azathioprine can induce myelosuppression, leukopenia, and, albeit less frequently, anemia and thrombocytopenia, as well as hepatitis and pancreatitis [46], requiring dose reduction or drug pausing. MMF is usually well-tolerated and therefore it has become a preferred antimetabolite agent. Next to gastrointestinal problems, MMF can lead to leukopenia dose-dependently [42], but its bone marrow suppressive effects are much lower than those of azathioprine. After heart transplantation, MMF in combination with calcineurin inhibitor therapy has been shown to be superior to azathioprine in preventing acute rejection episodes, development of coronary allograft vasculopathy, and mortality despite slightly higher infection rates [47, 48].

### Optimizing Immunosuppression Regimens

Here, we focused primarily on immunosuppressive compounds inhibiting T cell activation, proliferation, and polarization but several other promising targets exist to prevent rejection against xeno-cell transplantation [26]. The ultimate goal of post-transplantation management is to minimize immunosuppression and their complications without sacrificing the efficacy of the therapy. Combining multi-drug therapies may result in dose reduction without lowering the treatment efficacy and potentially leads to less frequent or diminished side effects. However, despite several randomized clinical trials seeking to optimize immunosuppression regimens, there is still no optimal and standardized immunosuppressive protocol. Optimal drug choice may vary between individuals and the choice of regimen is dependent on different factors such as efficacy, potential for drug interactions, and tolerability. Hence, also for preclinical studies, we should aim for high standards when performing immunosuppression.

## Preclinical Cardiac Cell Transplantation Studies

Survival of non-autologous cell grafts—and to that end also the possible efficacy—of non-autologous cardiac cell transplantation is dependent on the type, dose, or combination of immunosuppressive drugs. Therefore, it is important to evaluate currently used immunosuppression regimens for cell transplantation in preclinical large animal models. Here, we focused specifically on the use of “second-generation” cardiac cells in transplantation studies to induce cardiac repair upon myocardial infarction. Although a lot of variabilities are seen, we divided them into preclinical studies using mono- and multi-drug therapies as an immunosuppressive regimen (Tables 1 and 2).

**Table 1:** Preclinical animal studies using monotherapy for immunosuppression by xeno-cell transplantation

References				
Jansen of Lorkeers et al. 2015	Kawamura et al. 2012	Takehara et al. 2008	Ye et al. 2014	Zhu et al. 2018 (mono- vs. multi-)
Animal				
Pigs	Mini-pigs	Pigs	Pigs	Cynomolgus monkey
Infarct model				
90-min LAD occlusion	Ameroid constrictor LAD	90-min LAD occlusion	60-min LAD occlusion	Permanent ligation
No. of treated with cells				
8	6	9	Not clearly defined	10
Cell amount + type				
10 × 10^6 Human Sca-1^+^ CPCs	2.5 × 10^7 hiPS-CMs sheets	2 × 10^7 hCDCs	2 × 10^6 hiPSC-CMs alone hiPSC-CMs, -ECs,-SMC combined Cells combined in patch	1 × 10^7 hESC-CVPCs
Donor				
Xenogeneic	Xenogeneic	Xenogeneic	Xenogeneic	Xenogeneic
Administration (route + timing)				
Intracoronary infusion in PBS 4 weeks after IR	Transplantation of sheets 4 weeks after IR	Intramyocardial injection in basic fibroblast growth factor (bFGF)-incorporating hydrogel 4 weeks after IR	Intramyocardial injection or fibrin patches with IGF-1 15 min after IR	Intramyocardial injections in DMEM/F12 30 min after MI
Immunosuppression				
Cyclosporine A	Tacrolimus	Cyclosporine A	Cyclosporine A	Cyclosporine A
Levels of immunosuppression (per day)				
Day – 1, 800 mg Week 1, 2 × 400 mg Week 2–4, 2 × 200 mg	0.6 mg/kg	5 mg/kg	15 mg/kg	30–45 mg/kg
Weight (in kg)				
68.5 ± 5.4	20–25	Not reported	~ 13	6.5 ± 0.2
Start immunosuppression				
1 day before transplantation	5 days before transplantation	5 days before transplantation	3 days before transplantation	5 days before transplantation
Evaluation of immune suppression				
Yes, between 30 and 110 ng/L	Not reported	Not reported	Not reported	Not reported, but cyclosporin serum concentrations of 100–250 ng/mL
Duration of follow-up (post-injection)				
4 weeks	8 weeks	4 weeks	4 weeks	3 days, 28 days
Survival/engraftment				
No cells found back	After 2 weeks yes After 8 weeks almost none Poor engraftment	After 4 days yes After 4 weeks yes, but less Engraftment visible	Yes Engraftment rarely in native tissue	After 3 days almost none No engraftment
Functional improvement				
No	Yes	Yes	Yes, but only combined cells + patch group	No

**Table 2:** Preclinical animal studies using multi-drug therapies for immunosuppression by xeno-cell transplantation

Reference						
Chong et al. 2014	Liu et al. 2018	Romagnuolo et al. 2019	Shiba et al. 2016	Williams et al. 2013	Kawamura et al. 2017	Zhu et al. 2018 (mono- vs. multi-)
Animal						
Pig-tailed macaque	Pig-tailed macaque	Pigs	Cynomolgus monkey	Pigs	Mini-pigs	Cynomolgus monkey
Infarct model						
90-min LAD occlusion	180-min LAD occlusion	90-min LAD occlusion	180-min LAD occlusion	90-min LAD occlusion	Ameroid constrictor LAD	Permanent ligation
No. of treated with cells						
4	5	6	7	10	17	12
Cell amount + type						
1 × 10^9 hESC-CMs	750 × 10^6 hESC-CMs	1 × 10^9 hESC-CMs	4 × 10^8 MHC-matched iPSC-CMs (*n* = 5) MHC-mismatched iPSC-CMs (*n* = 2)	1 × 10^6 hCSCs (*n* = 5), 1 × 10^6 hCSCs +200 × 10^6 hMSCs (*n* = 5)	Amount of cells not reported. Cell sheet (*n* = 8) Cell sheets + omentum flap (*n* = 9)	1 × 10^7 hESC-CVPCs
Donor						
Xenogeneic	Xenogeneic	Xenogeneic	Allogeneic	Xenogeneic	Xenogeneic	Xenogeneic
Administration (route + timing)						
Intramyocardial injection in pro-survival cocktail 2 weeks after IR	Intramyocardial injection in pro-survival cocktail 2 weeks after IR	Intramyocardial injection in pro-survival cocktail 3 weeks after IR	Intramyocardial injection in pro-survival cocktail 2 weeks after IR	Intramyocardial injections in PBS 2 weeks after IR	Transplantation of sheets 4 weeks after MI	Intramyocardial injections in DMEM/F12 30 min after MI
Immunosuppression						
Cyclosporine A Methylprednisolone CTLA4-Ig	Cyclosporine A Methylprednisolone CTLA4-Ig	Cyclosporine A Methylprednisolone CTLA4-Ig	Tacrolimus Methylprednisolone	Cyclosporine A Methylprednisolone	Tacrolimus Prednisolone MMF	Cyclosporine A Methylprednisolone Basiliximab
Levels of immunosuppression (per day)						
Day −5-term: unknown dose of cyclosporine A, to reach serum levels of 200–250 μg/L Day −1: 500 mg methylprednisolone Week 1–2: 0.1–1.5 mg/kg methylprednisolone Day −1-term: 12.5 mg/kg CTLA4-Ig every 2 weeks	Day −5-term: unknown dose of cyclosporine A, to reach serum levels of 200–250 μg/L Day −1: 30 mg/kg methylprednisolone Day 0–1: 6 mg/kg methylprednisolone Day 2-term: 3 mg/kg methylprednisolone Day −1-term: 12.5 mg/kg CTLA4-Ig every 2 weeks	Day −5-term: 2 × 10–16 mg/kg cyclosporine A Day 0–14: 250 mg methylprednisolone with tamper to 125 mg Day 14-term: 125 mg methylprednisolone Day 0-term: 12.5 mg/kg CTLA4-Ig every 2 weeks	Day −2-term: 0.1 mg/kg tacrolimus Day −1-2: 10 mg/kg methylprednisolone Day 3-term: 1 mg/kg methylprednisolone	Day −2-term: 2 × 400 mg cyclosporine A Day 1: 250 mg methylprednisolone Day 2-term: 125 mg methylprednisolone	0.75 mg/kg tacrolimus 20 mg prednisolone 500 mg MMF	Day −5-term: 30–45 mg/kg cyclosporine A, to reach serum levels of 100–250 ng/mL Day −1: 500 mg methylprednisolone Day 0-term 1 mg/kg methylprednisolone Day 0–4: 10 mg basiliximab
Weight (in kg)						
8.6–12.3 kg	5.2–12.6 kg	20–30 kg	2.6–3.45 kg	35–40 kg	20–25 kg	6.4 ± 0.2
Start immunosuppression (before transplantation)						
5 days before transplantation cyclosporine 1 day before transplantation methylprednisolone and CTLA4-Ig	5 days before transplantation cyclosporine 1 day before transplantation methylprednisolone and CTLA4-Ig	5 days before transplantation cyclosporine On the day of transplantation methylprednisolone and CTLA4-Ig	1 day before transplantation methylprednisolone 2 days before transplantation tacrolimus	2 days before transplantation cyclosporine On the day of transplantation methylprednisolone	5 days before transplantation	5 days before transplantation cyclosporine A 1 day before transplantation methylprednisolone 2 h before transplantation basiliximab
Evaluation of immune suppression						
Not reported, but cyclosporin administration based on serum levels	Not reported, but cyclosporin administration based on serum levels	Not reported	Yes, day 84 tacrolimus levels reported	Yes, cyclosporine levels reported	Not reported	Not reported, but cyclosporin administration based on serum levels
Duration of follow-up (post-injection)						
14 days (*n* = 1) 28 days (*n* = 2) 84 days (*n* = 1)	28 days (*n* = 3) 84 days (*n* = 2)	4 weeks	4 weeks MHC-mismatched iPSC-CMs 12 weeks MHC-matched iPSC-CMs	4 weeks	3 months	3 days 28 days 140 days
Survival/engraftment						
Yes	Yes	Yes	MHC-matched iPSC-CMs yes, MHC-mismatched iPSC-CMs no	Yes	Yes	Yes, but not after 140 days
Functional improvement						
Variable responses	Yes	No	Yes	Yes	Yes	Yes

### Monotherapies

Preclinical studies using monotherapy with xeno-cell transplantation all applied calcineurin inhibitors, predominantly cyclosporine A [49–51] (Table 1). Treatment initiation and doses showed large variations and so do the outcomes. For example, human cardiosphere-derived cells (hCDCs) were transplanted alone and together with a basic fibroblast growth factor-incorporating hydrogel in the infarcted pig heart, while animals received cyclosporin treatment of 5 mg/kg/day [50]. Retention of hCDCs was significantly better when transplanted together with the hydrogel, but more importantly, they observed graft survival after 4 days and 4 weeks, engraftment of the transplanted cells in recipients resident tissue (based on the presence of human Y chromosomes), and functional improvement. In contrast, in a comparable pig model, human Sca-1+ cardiomyocyte progenitor cells (CPCs) could be found back: however, no functional improvement was observed 4 weeks post-infusion, while animals received a higher dose of CyA (day − 1, 800 mg; week 1, 2 × 400 mg; weeks 2–4, 2 × 200 mg) [49]. An even higher CyA dose (15 mg/kg/day) was used in a study from Ye et al., in which hiPSC-CM alone, hiPSC-CMs together with hiPSC-ECs and hiPSC-SMC, and in combination within a fibrin patch were transplanted in the post-infarcted pig heart [51]. This resulted in graft survival and engraftment although integration in the native tissue did not occur and functional improvement was restricted to the patch group only.

Next to the different graft types and treatment regimens, the route of graft administration (i.e., intracoronary vs. intramyocardial) might have caused the observed inter-study differences. However, previous studies have shown that engraftment does not depend on the administration route [52]. Interestingly, several studies, including the above, observed higher retention rates when cells are transplanted in patches or co-administered with hydrogels. When tacrolimus (0.6 mg/kg/day) was given to mini-pigs that received hiPSC-CM sheets post-MI [8], cell survival was observed 2 weeks post-transplantation and cardiac function was improved compared to control animals. However, almost no cell survival was seen after 8 weeks.

Altogether, these studies yet remain inconclusive concerning the efficacy of cyclosporine or tacrolimus to provide sufficient immunosuppression for xeno-cell transplantation applications as monotherapies.

### Multi-drug Therapies

Recent preclinical studies mostly use a multi-drug approach with a calcineurin inhibitor as one of the compounds (Table 2). As second drug, corticosteroids are chosen frequently [53, 54], and some studies add MMF [9], CTLA4-Ig, a drug for rheumatoid arthritis that blocks the co-stimulatory signal (T cell activation, signal 2) by binding both CD80 and CD86 [55–57] or basiliximab, a monoclonal antibody that targets the IL-2 receptor (T cell proliferation, signal 3) [58]. Although these studies were done in different animal models, including pigs, pig-tailed macaque, and cynomolgus monkeys, with different follow-up time, and different immunosuppressive drugs and doses of immunosuppression, all show survival of xeno-transplanted cells at termination of the experiment.

In two landmark papers of the Murry group [55, 56], pig-tailed macaques received intramyocardial injections of hESC-CM while being treated with cyclosporine A, methylprednisolone, and CTLA4-Ig. Extensive survival and remuscularization were seen in all macaques, with graft sizes ranging from 0.7 to 5.3% [55] and 1.1–3.4% of the left ventricle [56]. The lack of B and T cell accumulation around the hESC-CM grafts suggests that this specific combination and dose effectively prevent graft rejection [55]. Unfortunately, details of used dose regimens, at least for cyclosporine, were not clear. The notion of using this multi-drug approach is further supported by hESC-CMs transplantation in pigs (2× daily 10–16 mg/kg cyclosporine A, 10 mg/kg methylprednisolone with tampering to 5 mg/kg and 12.5 mg/kg CTLA4-Ig every 2 weeks) [57], which resulted in remuscularization with sparse immune cell infiltration.

One of the few studies comparing a mono- vs multi-drug approach was published in 2018 [58]. Here, hESC-derived cardiovascular progenitors (hESC-CVPC) were transplanted intramyocardially into the infarcted heart of cynomolgus monkeys receiving either cyclosporine A alone (30–45 mg/kg), or a combination of cyclosporine (30–45 mg/kg/day), methylprednisolone (1 mg/kg/day with loading dose of 500 mg), and basiliximab (1.5 mg/kg/day from day 1 till day 4). hESC-CVPC transplanted in animals receiving monotherapy treatment almost completely disappeared 3 days post-transplantation and the areas around the injection sites were dominated by immune cells. Upon combination therapy, hESC-CVPCs were still present at the site of administration with significantly less immune infiltration. Moreover, although less hESC-CVPC were detectable after 28 days, better recovery of left ventricular function and less apoptosis of native cardiac cells was seen in groups that received the multi-drug approach. However, no transplanted cells could be detected in either group after 140 days follow-up. Nevertheless, this study showed superior efficacy of multi-drug approaches compared to a monotherapy, albeit even the applied multi-drug regimen was not able to prevent long-term rejection.

Not all preclinical studies applied xeno-cell transplantation. Allogeneic iPSC-CM was transplanted in cynomolgus monkeys receiving a combination of tacrolimus (0.1 mg/kg/day) and methylprednisolone (day 1–3 10 mg/kg/day, from day 3 forward 1 mg/kg/day) [53]. Here, MHC-matched or MHC-mismatched iPSC-CMs were injected intramyocardially 2 weeks after IR. Animals receiving MHC-matched iPSC-CMs showed graft survival after completion of the follow-up period (12 weeks), with no evidence of immune rejection. In addition, MHC-matched iPSC-CMs improved contractile function and were structurally and electrically integrated into the heart. In contrast, MHC-mismatched iPSC-CMs were rejected and T cell infiltration was evident after 4 weeks of transplantation [53]. This has been confirmed by others after subcutaneous transplantation of MHC-matched iPSC-CM and tacrolimus monotherapy (2 mg/kg/day) or no immune-suppressive drugs applied. Here, a host immune response to the graft was still induced in the monotherapy group [59] and graft survival therefore be designated to the immunosuppressive regimen.

In summary, although most studies are difficult to compare due to the mentioned variations in treatment regimens and doses, the evidence suggests that xeno-cell survival and engraftment is more likely when applying a multi-drug immunosuppressive approach.

## Discussion/Recommendations

Cell-based therapies are intensively investigated to reduce morbidity and mortality associated with HF. “Second-generation” cardiac cell products have great therapeutic potential, but translation from preclinical studies towards clinical studies is hampered by several major barriers, including low retention, engraftment, and survival rates of transplanted cells. Novel approaches enhancing cell retention or cell delivery are elaborately investigated today. Still, immunological intolerance of cell transplants in preclinical studies poses a challenge. Although all preclinical cell transplantation studies using xenografts or allografts discussed in this review use some form of immunosuppression, unawareness of the necessity of immunosuppression still exists and there are no clear immunosuppression guidelines to date.

This is illustrated by the many different immunosuppressive regimens summarized in Tables 1 and 2. Mono- and multi-drug-therapies use various immunosuppressive compounds and different dosages, while triple immunosuppression after organ transplantation has been used in clinical practice for years. Targeting different pathways has been shown to yield better results and increase treatment efficacy while limiting adverse side effects. The study from Zhu et al. 2018, showed the strength of a multi-drug regimen over monotherapy use [58]. The combination of cyclosporine A, methylprednisolone, and basiliximab was more effective in terms of improved cell survival over 28 days compared to no survival with cyclosporine treatment only. Nonetheless, no cells survived after 140 days. To the best of our knowledge, this is the longest follow-up period for preclinical cell therapies so far. The limited efficacy of monotherapy as evidenced by the low survival also favors multi-drug approaches. Therefore, we recommend using multi-drug therapies for optimal immunosuppression.

Another issue concerns adequate dosing to reach and maintain therapeutic plasma concentrations. Immunosuppressive agents have distinct pharmacokinetic and pharmacodynamic profiles that affect both efficacy and tolerance. This stresses the importance to contemplate inter-species variability in pharmacokinetics and -dynamics. Hence, human doses of immunosuppressive compounds cannot simply be transferred one-to-one to other animal species. Already in 1988, it was shown that the same CyA and prednisolone doses per kilogram body weight resulted in significantly lower plasma concentrations in pigs than in humans [60]. In order to obtain comparable drug concentrations as in humans, cyclosporine doses needed to be doubled and prednisolone doses had to be 10-fold higher in pigs. This was even more pronounced for oral administration, where pigs required a 4–6-fold cyclosporin dose and a 31-fold higher prednisolone dose. These inter-species differences were attributable to an increased distribution volume, increased clearance, and reduced systemic availability due to incomplete absorption and first-pass metabolism in pigs [60]. As a result, proper immunosuppressive regimens for preclinical cell transplantation studies require perhaps different dosing to have functional effects. Determining the optimal dosing regimens from excising preclinical studies remains complicated as information regarding drug administration route (i.e., intravenous vs. oral) and plasma target levels is scarce. Hence, adequate reporting is essential. Higher doses also increase the likelihood of adverse side effects and toxicity which are usually not reported, probably due to the generally limited treatment duration, but should be evaluated. Furthermore, different responses to specific immunosuppressive compounds have been reported within the same species, e.g., in pigs. Despite administering the same CyA dose to all animals, plasma levels ranged from 30 to 110 ng/L [49], stressing the relevance of drug monitoring once more. Accordingly, we advise to first design a sufficient immunosuppressive regimen in the envisioned model. This then needs to be evaluated in a pilot study where the regimen efficacy is demonstrated (e.g., by in vivo assays focusing on immune cell infiltrates in easily accessible areas (subcutaneous)) and should be reported accordingly. As immunosuppressive regimes may also affect the disease itself, proper controls (e.g., diseased animals treated with immunosuppressive agents alone) should be included to evaluate their outcome on disease. In addition, we recommend to carefully evaluate and perform close plasma concentration monitoring of immune suppressants when performing preclinical animal experiments.

In conclusion, due to the lack of clear guidelines for immunosuppressive regimens, preclinical studies show substantial variability in the use of immunosuppression (compounds used, administration route, and dosage). Applying immunosuppression without careful evaluation for efficacy in the model of interest increases the risk of translational failure. This calls for generalized and high-quality standards for immunosuppression when performing preclinical cell transplantation studies. Our recommendations for designing proper immunosuppressive regimens include using a multi-drug approach of which the efficiency is demonstrated for the model of interest and perform close plasma concentration monitoring of immune suppressants during preclinical animal experiments.
